# Pulmonary arterial hypertension in critically ill elderly patients

**DOI:** 10.12669/pjms.331.10913

**Published:** 2017

**Authors:** Yun-yun Zhang, Fan Xu, Ming Chu, Li-qing Bi

**Affiliations:** 1 Yun-yun Zhang, Department of Geriatric Intensive Care Unit, Jiangsu Province Hospital, Nanjing, 210029, China; 2 Fan Xu, Department of Geriatric Intensive Care Unit, Jiangsu Province Hospital, Nanjing, 210029, China; 3 Ming Chu, Department of Geriatric Intensive Care Unit, Jiangsu Province Hospital, Nanjing, 210029, China; 4 Li-qing Bi, Department of Geriatric Intensive Care Unit, Jiangsu Province Hospital, Nanjing, 210029, China

**Keywords:** Critically ill patients, Pulmonary arterial hypertension, The elderly, Risk factors

## Abstract

**Objective:**

To assess the incidence, possible risk factors and prognosis of pulmonary arterial hypertension (PAH) in critically ill elderly patients.

**Methods:**

We selected 122 cases admitted to the ICU, ages 60–93 years old. An echocardiography examination was performed within four days after admission to the ICU. PAH is usually suspected if the patient’s pulmonary artery systolic pressure ≥ 40 mmHg. We collected echocardiography data, relevant clinical data and routine laboratory data; we then used a statistical method to analyze the risk factors for PAH in critically ill elderly patients and examined its impact on the prognosis.

**Results:**

Total 51 patients were diagnosed with PAH. The prevalence of critically ill elderly patients with PAH was 41.8%. The ANOVA analysis showed that if patients had COPD (*P* = 0.031) and/or respiratory failure (*P* = 0.021), they were more prone to PAH. An enlarged left atrium (*P* = 0.038) and/or right ventricle (*P* = 0.029), a declining left ventricle fractional shortening rate (*P* = 0.038), and an elevated amount of the brain natriuretic peptides (*P* = 0.046) were all associated with the occurrence of PAH. Multivariate regression analysis showed that the left atrial diameter (*P* = 0.045) was the risk factor in critically ill elderly patients with PAH. The 30-day mortality rate was 33.3% for elderly patients with PAH, which is statistically significant (*P* = 0.035) when compared with the mortality rate of patients with normal pulmonary artery pressure. Our multivariate regression analysis also showed that, for critically ill elderly patients admitted in the ICU, PAH (*P* = 0.039) is risk factor for increased mortality.

**Conclusions:**

A higher incidence of PAH occurs in critically ill elderly patients. PAH is more likely to occur in patients with an enlarged left atrium, and these problems adversely impact the prognosis.

## INTRODUCTION

Pulmonary arterial hypertension (PAH) is a progressive disease that is often fatal. PAH occurs when the pulmonary circulation pressure is higher than normal, a condition caused by diseases (such as heart or lung disease and lung artery disease), hereditary factors and a variety of pathogens. The clinical characteristic is that pulmonary vascular resistance progressively increases, which obstructs the right ventricular ejection, leading to right heart failure and even death.^1^ PAH is an important pathophysiological link that occurs during the development of many heart and lung diseases. Once it occurs, it seriously affects the progress and prognosis of primary cardiopulmonary disease, thus making it a key global health issue.^2^

PAH used to be considered a disease occurring mainly in young people. However, further research has found that the prevalence and mortality of PAH are on the rise in the elderly population.^3^,^4^ The pathogenesis of PAH is closely related to heart and pulmonary circulation. PAH etiology, clinical characteristics and treatment in the elderly are very different from those for younger patients, largely due to aging as the heart and lung function decline. In fact, elderly patients with PAH are often admitted to the intensive care unit (ICU) for treatment because of the disease’s progression and right ventricular dysfunction. Unfortunately, there is currently no research about the prevalence, risk factors and outcomes in critically ill elderly patients with PAH. Right heart catheterization is the gold standard for PAH diagnosis, but the examination is an invasive procedure, especially for elderly patients with poor adherence, and is not suitable for promotion as a clinical screening.

In 2009, the European Society of Cardiology (ESC) released its PAH diagnosis and treatment guidelines, which clearly stated that transthoracic Doppler echocardiography is a good non-invasive screening method for PAH.^5^ We observed critically ill elderly patients for this study. We used color Doppler ultrasound for the preliminarily screening of the PAH patients; we then collected clinical data and investigated the prevalence, prognosis and possible potential risk factors of PAH. We provide evidence in this paper for the prevention and treatment of critically ill elderly patients with PAH.

## METHODS

### Subjects

In this study, a total of 122 elderly patients were included from January 2014 to March 2015; these patients had been admitted to the geriatric ICU, comprehensive ICU, emergency ICU and respiratory ICU of Jiangsu Provincial Hospital. There were 78 male patients and 44 female patients; their ages ranged from 60–93 years old, with an average age of 74.4 (± 8.0) years old. All the patients met the criteria for admission into the ICUs according to the Intensive Medicine and Constructive Management Guide, 2009 Edition. This study was approved by the ethics committee of Jiangsu Province Hospital with the reference number: 2016-SR-148.

### Methods

We performed an echocardiography examination on each patient within the first four days of their ICU admission. In addition to the conventional measurement of the diameters of the four heart cavities, we recorded, in detail, the related echocardiography data (i.e., the pulmonary artery systolic pressure (PASP) and left ventricular ejection fraction (EF), as well as the indicators reflecting the systolic and diastolic function of the right and left ventricles). We collected clinical data, routine laboratory data and a detailed record of each patient’s medical history, including their use of medication. We recorded their lipids, N-terminal pro-brain natriuretic peptides, total bilirubin, uric acid and high-sensitivity C-reactive protein. We recorded each patient’s prognosis and outcome, as well as any treatments provided to the patient during the ICU stay, including the use of mechanical ventilation and vasoactive drugs. Patients were divided into a normal pulmonary arterial pressure group and a PAH group in accordance with the internationally recommended standard for PAH diagnosis by echocardiography (PASP ≥ 40 mmHg).^6^ Our statistics for the PAH group were categorized according to WHO classification criteria based on the patient’s medical history and test data. Statistical methods were used to compare the differences in the clinical data for issues such as cardiac ultrasound data and the laboratory data between the two groups. We used 30-day mortality and length of stay in ICU as the main outcome variable and analyzed the risk factors for PAH occurrence in critically ill elderly patients. In addition, all cases were grouped according to the 30-day mortality, and we used single factor and multivariate regression analysis to analyze the impact of the patient’s primary disease and PAH on the prognosis.

### Statistical Methods

We used SPSS 21.0 software for all statistical analysis; χ^2^ test was used for categorical data, and numerical data were presented as x̄ ± s and analyzed with *t* test. Logistic regression analysis was used. *P* < 0.05 was considered statistically significant.

## RESULTS

### Clinical Data Analysis

Using the estimated PASP data, 122 cases were divided into two groups: a normal pulmonary artery pressure group and the PAH group. The PAH group contained 51 cases (41.8%) and the average PASP was 51.5 ± 12.9 mmHg; when this average was compared to the normal pulmonary group, the difference was found to be statistically significant (*P* < 0.0001). Based on the patients’ history and test data, 17 cases (33.3%) were categorized as Group 2 (that is, they suffered from pulmonary hypertension caused by left heart disease) and 26 cases (51.0%) were categorized as Group 3 (they suffered from pulmonary hypertension due to chronic hypoxic disease); these data were classified according to the revised diagnostic classification criteria released from the WHO Fifth World Symposium on Pulmonary Hypertension held in Nice, France, in 2013. The remaining 8 cases could not be clearly classified according to the existing data. The PAH group was compared with the normal pulmonary artery pressure group based on clinical indicators such as age, sex, current illness, length of stay in ICU, 30-day mortality and echocardiographic data, as well as part of the laboratory data (Tables I and II). The results showed that COPD and/or respiratory failure were more likely to co-exist with PAH (χ^2^ = 4.626, *P* = 0.031; χ^2^ = 5.358, *P* = 0.021). The 30-day mortality was 33.3% for the PAH group, which was higher than that for the normal pulmonary artery pressure group (χ^2^ = 4.423, *P* = 0.035). The diameter of the left ventricle, the right ventricular end-diastolic diameter and the amount of N-terminal pro-brain natriuretic peptides for the PAH group were higher than those found in the normal pulmonary artery group (*t* = 1.53, *P* = 0.038; *t* = 2.801, *P* = 0.029; *t* = 1.519, *P* = 0.046), while the LV fractional shortening rate was lower for the latter group (*t* = −0.456, *P* = 0.038).

**Table-I:** Age, sex, current illness, outcome variable and other clinical indicators of the two groups.

	Pulmonary arterial hypertension group	Normal pulmonary artery pressure group	χ^2^	P
Number of cases	51 (41.8%)	71 (58.2%)		
PASP (mmHg)	51.5 ± 12.9	30.4 ± 5.4		<0.0001
Age (years)	73.9 ± 8.4	74.8 ± 7.8		0.462
Sex (male)	28 (54.9%)	50 (70.4%)	3.101	0.078
Hypertension (cases)	24 (47.1%)	36 (50.7%)	0.158	0.691
Diabetes (cases)	11 (21.6%)	20 (28.2%)	0.682	0.409
COPD (cases)	19 (37.3%)	14 (19.7%)	4.626	0.031
Septic shock (cases)	17 (33.3%)	25 (35.2%)	0.046	0.83
Cardiac-related coronary heart disease (cases)	11 (21.6%)	22 (30.9%)	1.334	0.248
Lung infection (cases)	25 (49.0%)	36 (50.7%)	0.034	0.854
Respiratory failure (cases)	24 (47.1%)	19 (26.8%)	5.358	0.021
Cerebrovascular diseases (cases)	5 (9.8%)	9 (12.7%)	0.241	0.623
Digestive system related (cases)	5 (9.8%)	10 (14.1%)	0.504	0.478
Renal failure (cases)	6 (11.8%)	7 (9.9%)	0.113	0.737
Length of stay in ICU (days)	17.0±14.6	13.8±9.4		0.090
30-day mortality (cases)	17 (33.3%)	12 (16.9%)	4.423	0.035

**Table-II:** Ultrasound data and some laboratory data of the two groups.

	Pulmonary arterial hypertension group	Normal pulmonary artery pressure group	t	P
Number of cases	51 (41.8%)	71 (58.2%)		
EF (%)	61.9 ± 7.5	60.8 ± 7.2	0.685	0.99
LAD (mm)	40.0 ± 9.9	37.6 ± 7.2	1.53	0.038
LVDd (mm)	47.5 ± 7.6	47.3 ± 6.7	0.054	0.298
LVDs (mm)	32.2 ± 6.4	31.4 ± 5.9	0.392	0.448
LVPW (mm)	9.8 ± 1.1	10.4 ± 1.3	−2.006	0.078
IVS (mm)	10.2 ± 1.3	10.6 ± 1.6	−1.535	0.097
FS (%)	32.9 ± 6.9	33.4 ± 4.0	−0.456	0.038
RVDd (mm)	41.5 ± 7.1	31.8 ± 4.2	2.801	0.029
NT-proBNP (pg/ml)	4279.25 ± 7505.48	2483.26 ± 5423.29	1.519	0.046
Uric acid (μmol/l)	270.32 ± 164.19	254.29 ± 143.54	1.329	0.06
Hypersensitive CRP (mg/L)	66.17 ± 70.72	85.43 ± 101.23	−0.774	0.155
Total bilirubin (μmol/L)	16.67 ± 26.19	16.13 ± 18.73	0.837	0.909

*Note:* EF: Ejection fraction; LAD: Left atrial diameter; LVDd: Diastolic left ventricular diameter; LVDs: Systolic left ventricular diameter; LVPW: Left ventricular wall thickness; FS: (LV) Fractional shortening rate; RVDd: Right ventricular end-diastolic diameter; NT-proBNP: N-terminal pro-brain natriuretic peptide; CRP: C-reactive protein.

### Multivariate Regression Analysis

We used logistic regression analysis for the statistically significant indicators obtained by univariate analysis between the normal pulmonary artery pressure group and the PAH group. When controlling for these clinical features, enlarged left atrium and right ventricle remained independently associated with PAH in critically ill elderly patients (*OR* = 0.806, 95% *CI*: 0.646–1.005, *P* = 0.045; *OR* = 0.735, 95% *CI*: 0.559–0.966, *P* = 0.027) (Table-III).

**Table-III:** Multivariate regression analysis of risk factors in critically ill elderly patients with pulmonary arterial hypertension.

Factors	B	SE	Wald χ^2^	P	OR	95% CI
COPD	0.220	1.264	0.030	0.862	1.246	0.105–14.836
Respiratory failure	−0.760	1.148	0.438	0.508	0.468	0.049–4.439
RVDd (mm)	−0.308	0.139	4.874	0.027	0.735	0.559–0.966
LAD (mm)	−0.216	0.113	3.675	0.045	0.806	0.646–1.005
FS (%)	−0.346	0.201	2.970	0.085	0.707	0.477–1.049
NT-proBNP (pg/ml)	0.000	0.001	0.454	0.500	1.000	0.998–1.001

### Analysis of Risk Factors for 30-day mortality

All cases were grouped based on 30-day mortality (Table-IV); the indexes were compared between the two groups, such as age, sex, current illness and whether the patient was provided with mechanical ventilation and/or vasoactive drugs when admitted to the ICU. The results of the logistic regression analysis showed that when critically ill elderly patients were admitted to the ICU, those who suffered from PAH, required the use of mechanical ventilation, or had an unstable blood pressure that needed vasoactive drug support were at an increased risk of mortality (*OR* = 2.513, 95% *CI*: 1.045–6.041, *P* = 0.039; *OR* = 3.479, 95% *CI*: 1.345–9.000, *P* = 0.010; *OR* = 3.226, 95% *CI*: 1.353–7.691, *P* = 0.008, respectively) (Table-V).

**Table-IV:** Age, sex, current illness and other clinical indicators of the two groups.

	Death group	Survival group	χ^2^	P
Number of cases	29 (23.8%)	93 (76.2%)		
Age (years of old)	76.24 ± 8.09	73.73 ± 7.87		0.12
Sex (male)	18 (62.1%)	60 (64.5%)	0.057	0.811
PAH (cases)	17 (58.6%)	34 (36.6%)	4.423	0.035
Lung infection (cases)	15 (51.7%)	46 (49.5%)	0.045	0.832
Hypertension (cases)	16 (55.2%)	44 (47.3%)	0.547	0.46
Cardiac-related Coronary heart disease (cases)	8 (27.6%)	25 (26.9%)	0.006	0.941
COPD (cases)	9 (31.0%)	24 (25.8%)	0.306	0.58
Diabetes (cases)	6 (20.7%)	25 (26.9%)	0.447	0.504
Cerebrovascular diseases (cases)	3 (10.3%)	11 (11.8%)	0.048	0.827
Digestive System Related (cases)	3 (10.3%)	12 (12.9%)	0.134	0.714
Renal failure (cases)	4 (13.8%)	9 (9.7%)	0.641	0.423
Whether mechanical ventilation used (cases)	16 (55.2%)	24 (25.8%)	8.651	0.003
Whether vasoactive drugs used (cases)	17 (58.6%)	25 (26.9%)	9.865	0.002

**Table-V:** Multivariate regression analysis: Mortality of critically ill elderly patients in the ICU.

Factors	B	SE	Wald χ^2^	P	OR	95% CI
Pulmonary arterial hypertension	0.922	0.448	4.24	0.039	2.513	1.045–6.041
Mechanical ventilation	1.247	0.485	6.61	0.01	3.479	1.345–9.000
Vasoactive drugs	1.171	0.443	6.979	0.008	3.226	1.353–7.691

## DISCUSSION

There are still many problems with treating those suffering from PAH due to the complexity of the disease and a lack of understanding of the pathogenesis of PAH. The typical patient’s prognosis remains poor and treatment options are limited, which poses a significant threat to the overall patient care. PAH patients require better treatment methods that incorporate all of the clinical characteristics of the elderly patients. Because of the special physiological mechanisms at work in the elderly, the harm from PAH is far greater for this population than that for younger people. RAVEAL research findings from a multi-center study found that male patients with PAH who were over 60 years of age had a higher mortality rate than those under 60 years of age.^7^ COMPERA found that the 1-, 2- and 3-year survival rates of elderly patients with a primary diagnosis of PAH were even lower than those in younger patients.^8^ However, there is still a lack of studies on the incidence of PAH, especially for critically ill elderly patients. The results of this study showed that the incidence of PAH in critically ill elderly patients is 41.8%, while studies from Cao^9^ and Rich^10^ reported that the incidence of PAH in elderly patients in general wards were 10.5% and 28.2%, respectively. Our data proved that elderly patients in the ICU had a significantly higher incidence of PAH than those in the general ward. This finding deserves more attention. Studies have found that the most common type of PAH in the elderly is the type associated with left heart diseases, particularly left atrial or ventricular heart disease.^4^ Grigioni et al.^11^ analyzed 196 cases of patients with class III–IV heart failure; after they adjusted for the clinical and laboratory examinations and other indicators, they found that PAH is an independent predictor of acute heart failure and cardiac death. Our data suggested that among the critically ill elderly patients admitted to the ICU, one-third of those with left ventricle diseases had PAH, while PAH caused by a chronic hypoxic pulmonary disease (such as COPD) were still the largest fraction (they accounted for half of the cases). This differs with the data from the general ward. Some studies showed that regardless the severity of PAH, the survival rate is low if COPD is the cause.^12^ In fact, if PAH progresses, it usually leads to an increase in the right heart load, resulting in right ventricular failure; this insight requires that healthcare providers pay more attention to the right ventricular function in critically ill elderly patients.

Our results showed that if COPD and respiratory failure are present, critically ill elderly patients are more likely to have PAH. An enlarged left atrium and/or right ventricle, declining left ventricle fractional shortening rate and an elevated amount of brain natriuretic peptides were all associated with the occurrence of PAH. Multivariate regression analysis showed that an increased left atrium diameter and a right ventricular end-diastolic diameter remained independently associated with PAH in critically ill elderly patients. In fact, an enlarged left atrium often suggested left ventricular diastolic dysfunction.^13^ Increased pressure inside the left atrium causes the pulmonary venous pressure to become elevated, resulting in a hardening of the distal pulmonary arteries, and ultimately, pulmonary artery remodeling. PAH can lead to restrictions in pulmonary blood flow, and characteristic progressive elevations in pulmonary vascular resistance. The increased afterload is the primary cause of right ventricular adaptation.^14^ So we considered the right ventricular hypertrophy was a consequence rather than an indicator of PAH, while the enlarged left atrium was the risk factor for PAH in critically ill elderly patients. Our study did not show that left ventricular systolic dysfunction was a risk factor for PAH occurrence. This lack of finding is probably related to the small sample size and sample constitution (Class 3 PAH sufferers account for half of the cases). However, to a certain extent, our study showed that there is some association between left ventricular diastolic function and the occurrence/development of PAH in critically ill elderly patients. Of course, this work will require a follow-up study to gather and analyze more detailed echocardiographic data.

We found that the 30-day mortality rate of critically ill elderly patients with PAH was 33.3%, higher than that of patients without PAH—the difference is statistically significant. This number is a little higher than that found in the study results of Huynh et al.^15^; the study subjects in this work were mainly PAH patients admitted to the ICU who were under 60 years of age. Other researchers have reported a connection between elevated pulmonary vascular resistance and early mortality.^16^ For the Group 3 PAH patients, the pulmonary circulation pressure in the early stages was already higher than that found in other types of PAH patients, which is consistent with the results of this study. Taking into account the variety of diseases often associated with ICU patients, we put factors such as current illness and the use of mechanical ventilation and vasoactive drugs upon admittance to the ICU into the multivariate regression analysis to determine the mortality rate. Our results showed that, when admitted to the ICU, patients with other diseases and PAH have an independent risk factor for increased mortality. The presence of respiratory failure and shock in critically ill patients, who often need mechanical ventilation and vasoactive drug treatment, suggests a poor prognosis for them. Huynh et al.’s^15^ study indicated that if PAH patients received CPR, their mortality rate was 100%. Mortality also increased if hemodialysis and mechanical ventilation were needed. At present, there are few studies regarding the outcome and prognosis of PAH patients in the ICU. A follow-up study is needed to confirm whether PAH interventions can improve a patient’s prognosis.

In summary, a higher incidence of PAH occurs in critically ill elderly patients; an enlarged left atrium is the risk factor for PAH in this group. There is some association between the left ventricular diastolic function and occurrence/development of PAH for this elderly population, as well. PAH is an independent risk factor for increased mortality in critically ill elderly patients. At present, further study is required to expand the sample size and study the pathogenesis and treatment interventions in order to improve the prognosis for those who suffer from PAH.
